# Basal ganglia stroke due to mild head trauma in pediatric age - clinical and therapeutic management: a case report and 10 year literature review

**DOI:** 10.1186/1824-7288-37-2

**Published:** 2011-01-06

**Authors:** Alessandro Landi, Nicola Marotta, Cristina Mancarella, Daniele Marruzzo, Maurizio Salvati, Roberto Delfini

**Affiliations:** 1 Department of Neurosurgical Sciences, Neurosurgery, University of Rome "Sapienza"-Rome, Italy; 2 Department of Neurosurgery, INM Neuromed, Pozzilli (IS), University of Rome "Sapienza"-Rome, Italy

## Abstract

Ischemia of the basal ganglia as an immediate consequence of minor head injury in children is rare (< 2% of all ischemic stroke in childhood) and is due to vasospasm of the lenticulostriate arteries. The clinical history of these lesions is particularly favourable because they are usually small, and also because the facial-brachial-crural hemiparesis typical of this pathology usually regresses after a period ranging from several weeks to several months, despite the persistence of an ischemic area on MRI. This is due to the well known neuronal plasticity of the CNS, in particular, of the primary motor cortex. The most effective therapeutic approach appears to be the conservative one, although the best treatment regimen is still not well defined.

Young patients should be closely monitored and treated conservatively with osmotic diuretics to reduce perilesional edema. At the same time, however, it is very important to exclude, by means of instrumental and laboratory studies, conditions that could favour the onset of ischemia, including emboligen heart disease, thrombophilia and acute traumatic arterial dissections. Generally speaking, the prognosis in these cases is good. The authors describe their experience treating a 10-month old baby girl, with a left lenticular nucleus ischemia and report a literature review.

## Introduction

Ischemia of the basal ganglia as an immediate consequence of minor head trauma in children under 18 months of age is a rare eventuality (< 2% of all ischemic stroke in childhood) [1], it is caused by vasospasm of the lenticulostriate arteries, frequent in childhood, which are disrupted by head injury. Generally, it manifests with nausea, vomiting, hemiparesis and drowsiness, a clinical picture known as JHTS syndrome (Juvenile Head Trauma Syndrome) [2]. We report the case of a 10 month old child who presented an acute ischemic lesion of the left lenticular nucleus, after a minor head trauma. The ensuing facio-brachio-crural hemiparesis improved over time until complete regression of symptoms 45 days later.

## Materials and methods

### Case description

**Figure 1:**
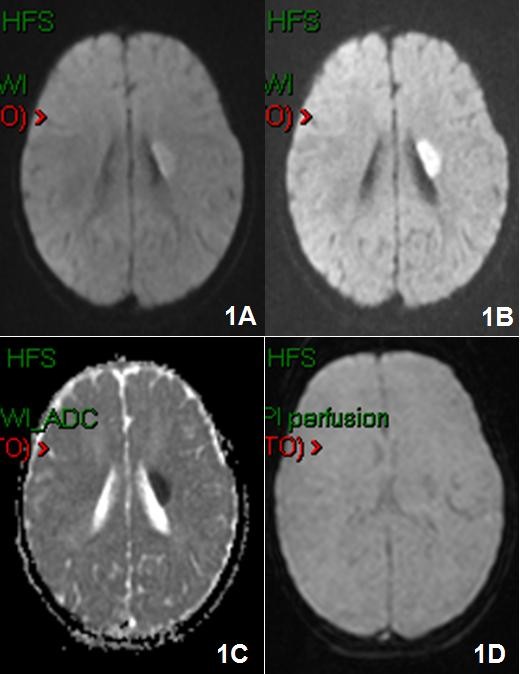
**Brain MRI with Gd-DTPA was performed and showed the presence of an hyperacute ischemic lesion of about 2 cm of diameter, in the left lenticular nucleus extended to the internal capsule 1A), 1B), 1C) in DWI and 1D) perfusion weighted sequences**.

A 10 month old girl with not clinical history of seizures was brought to our observation following a head injury involving the right temporo-occipital region: trauma had been caused by an accidental fall from a changing table at height of 1 m. She didn't lose consciousness but developed a right hemiparesis after 15 minutes. At neurological examination the GCS score was 14, and the child was isochoric with isocyclic pupils: there was a right facio-brachio-crural hemiparesis with muscle strength of -3/5. A head CT scan, done in emergency, showed a right occipital bone fracture with moderate diastases of the right branch of the lambdoid suture, without any intra or extraaxial blood collections. A brain MRI with Gd-DTPA was performed and documented the presence of a hyperacute ischemic lesion of about 2 cm in diameter, in the left lenticular nucleus extending to the internal capsule (Figure 1).

**Figure 2:**
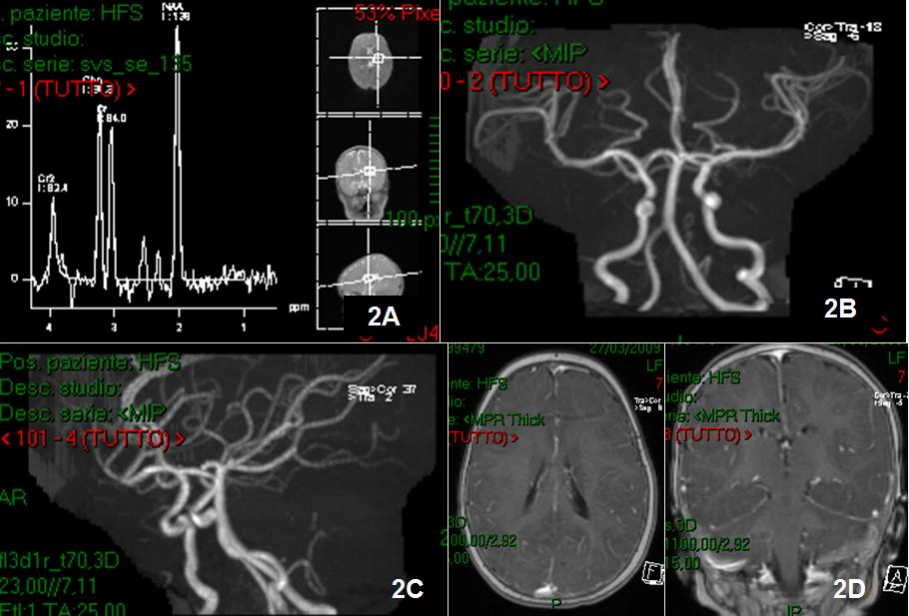
**MRI spectroscopy (2A), angio-MRI sequences (2B, 2C), and T1 weighted MRI with Gd-DTPA (2D)**. showed anterior, posterior circulation and circle of Willis vessel's perviety

**Figure 3:**
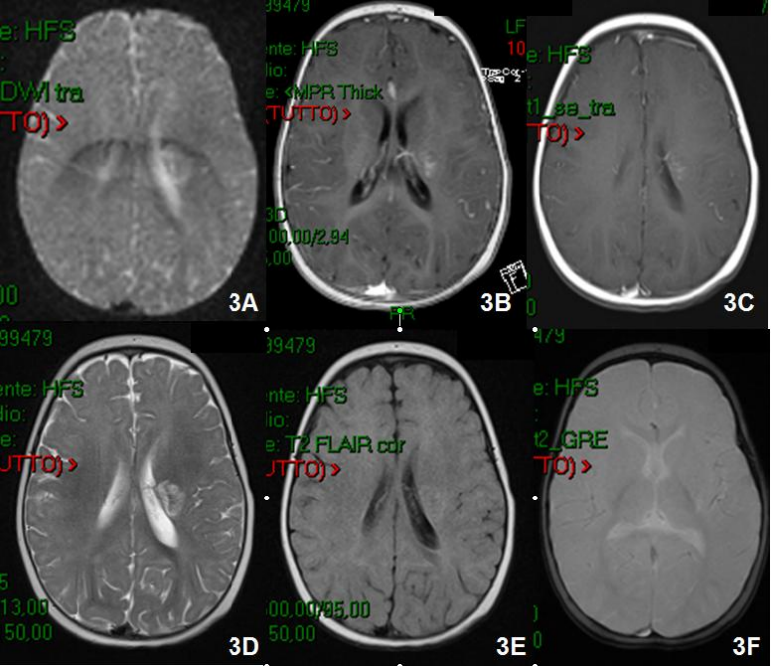
**Brain MRI with Gd-DTPA performed 45 days after the first one showed a reduction of the left lenticular nucleus lesion**.

Since a suspicion of acute arterial dissection existed, the investigation was completed with angiography sequences showing anterior and posterior circulation and perviety of the vessels of the circle of Willis (Figure 2). To rule out the possibility of an emboligen heart disease or thrombophilia, urgent haematological and cardiological videats were performed together with Doppler ultrasound evaluatin of neck vessels and echocardiography to exclude pathological findings. The suspicion of congenital thrombophilia, prompted us to run a complete and specific blood workup: dosage of VIII, IX, XI, XIII factors, lipoprotein A and homocysteine which were all within normal range. We evaluated possible gene mutations of factor V Leiden and of the MTHFR which were heterozygous and the CANAIA gene coding for a structural protein of Ca channel that was heterozygous. The prothrombin 20210 A did not show mutations. Platelet count, PT, PTT, Protein C, and D-dimer assays were negative. Anti-nuclear and anti ds-DNA antibody were evaluated and proved negative similarly to IgM and IgG anticardiolipin and IgM and IgG antiphospholipid dosage. We also executed thyroid hormone assays that were normal. The child immediately underwent treatment with oral anticoagulants (calciparine 80 U/kg until the exclusion of coagulopathy) and osmotic diuretics (mannitolo 0,25 g/kg, 4 times a day until the resolution of the perilesional edema). A new brain MRI was performed 12 hours later which showed a stable situation. A progressive improvement of the right hemiparesis was observed from the first day of admission, but not of the facial paresis. After 45 days all symptoms had disappeared and a brain MRI with GD-DTPA only showed the sequelae of the left capsular lenticular ischemia (Figure 3).

### Literature review

A review of the literature was conducted using Pubmed. The search was undertaken using the terms "mild head trauma", "child", "basal ganglia ischemia". References from the retrieved reports were checked to identify other possible reports. We selected manuscripts reporting young patients from the age of 0 to 18 months presenting lenticulo-capsular ischemia due to a mild head injury. The data extracted included, number of patients, male/female ratio, age distribution, location of the lesions, treatment, and clinical outcomes (Table 1) [1,3-5].

**Table 1:** Historic Cohor: Treatment, Localization and Clinical Outcomes.

Patient	Age/Gender	First Symptom	Lesion Location (CT Scan, MRI or Both)	Treatment	Clinical Outcome (Time of Follow-up in Months)	Risk Factors
**1[3]**	1.5/M	Hemiparesis	Internal capsule	Conservative	Good/13	None
**2[3]**	1.5/M	Hemiparesis	Lentiform nucleus	Conservative	Good/13	None
**3[4]**	1/M	Left-sided hemiparesis	left internal capsule and corona radiata	Conservative	Good/1	None
**4[1]**	1/F	Left-sided hemiparesis	Right lentiform nucleus and corona radiate	Conservative	Good/3	None
**5[1]**	1/M	Left-sided hemiparesis	Right lentiform nucleus and corona radiate	Conservative	Good/3	None
**6[1]**	1.2/F	Left-sided hemiparesis	Right basal ganglia and corona radiata	Conservative	minimal pyramidal tract signs in her left side/1	None
**7[5]**	11/M	left upper motor neuron 7th nerve palsy and left hemiparesis	bilateral basal ganglia, internal capsule and periventricular white matter	Conservative	mild dysarthria and bilateral extensor plantar/12	None
**8[5]**	1/M	Right hemiparesis	in left basal ganglia and internal capsule	Conservative	Good/3	None
**9[5]**	18/M	Right hemiparesis	left basal ganglia and internal capsule	Conservative	Good/5	born out of 2nd degree consanguinity

## Results

**Figure 4:**
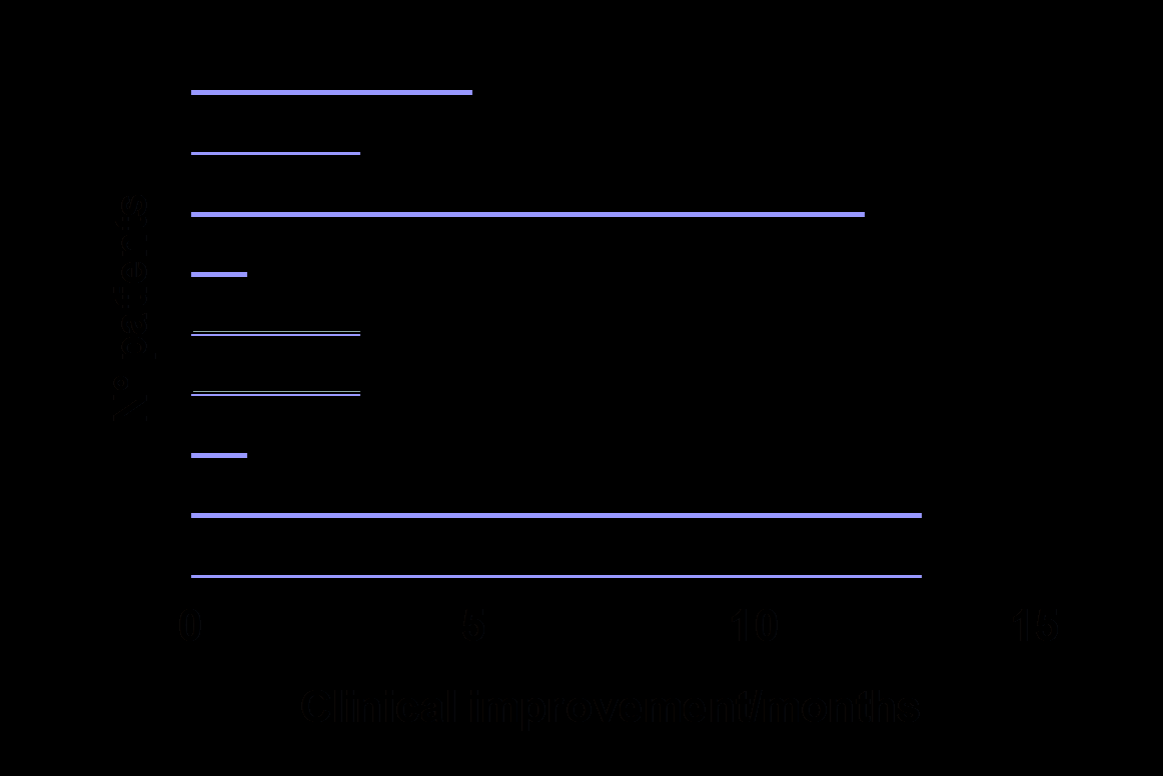
**Clinical improvement during follow up (months in the abscissa) in the patients studied in the literature review, treated conservatively**.

From 2000 to 2010, nine patients were found in the literature, 7 males and 2 females. All of them were studied by brain MRI that showed ischemia involving the left internal capsule and corona radiate ischemia in 3 cases, the right lentiform nucleus and the corona radiate in 3, the bilateral basal ganglia, internal capsule and periventricular white matter in 1, the internal capsule in 1 and the lentiform nucleus in 1 (the side in the last 2 cases was not specified). Hemiparesis occurred on the side opposite to the ischemia. All patients were treated conservatively, but the type of drugs used were not described in detail in any of the reports. All of them made a clinical improvement in less than 13 months (Figure 4). All of the patients underwent radiological follow up with Gd-DTPA MRI that always showed radiological evidence of the lesion. Hence, 7 patients were symptom-free and in 2 cases there was an improvement of the clinical status without complete resolution. None of the patients had associated risk factors.

## Discussion

To classify the mild head injury in the case described here, we took into account the guidelines of the Mild Traumatic Brain Injury Interdisciplinary Special Interest Group of the American Congress of Rehabilitation Medicine. These state that mild head trauma is a condition in which there is:

1 - loss of consciousness not exceeding 30 minutes;

2 - GCS between 13 and 15 after 30 minutes from the trauma;

3 - post-traumatic amnesia not exceeding 24 hours [6].

In childhood, cerebral infarction due to a mild head trauma is a rare eventuality, with an estimated incidence of about 1,9% [1]. Despite the rarity of the event, in children less than 18 months old the occurrence of this condition causes ischemia of the basal ganglia and, therefore, facio-brachio-crural hemiparesis, which frequently undergoes remission in a period ranging from one week to several months (usually 3 months) [4]. Secondary causes responsible for cerebral ischemic lesions in children below 18 months are attributable to traumatic dissection of the common carotid, internal carotid arteries or of the vessels of circle of Willis, congenital predisposition to rupture of cervical or intracranial arteries, emboligen heart disease and congenital thrombophilia [7,8]. In this case, before classifying a cerebral infarction in children below 18 months of age as idiopathic, it is imperative to exclude all possible secondary causes.

In cases similar to the one we describe here, it is necessary to exclude a possibility of surgical treatment of the lesions primarily responsible for symptoms such as bleeding, so the first investigation to perform is a CT scan. Because children are more sensitive to radiation, we prefer not to have a CTA study, but CT scan alone does not show hyperacute ischemic lesions. In this case young patients have to be submitted to:

2 - Brain MRI with Gd-DTPA which shows, when there is a cerebral infarction, hypointense image on T1 and hyperintense on T2, FLAIR and DWI corresponding to the ischemic area. 3 - Angio-MRI to rule out any congenital or anatomical abnormalities due to the trauma of the circle of Willis, responsible for ischemia. Although the gold standard for this purpose remains angiography, we think Angio-MRI should be the first study to be performed after CT scan.

4 - echocardiogram to exclude emboligen heart disease and echocolordoppler to exclude a traumatic carotid dissection.

5 - complete blood work-up in suspected congenital thrombophilia.

In the event of an idiopathic ictus cerebri in a child < 18 months of age after a mild head trauma, the aetiology is due to the particular anatomical characteristics of the arteries and of the brain parenchyma at this age, especially the lenticulostriate arteries, terminal branches of ACM responsible for the flow to caudate, putamen, internal capsule and pallidus. These vessels create an acute angle with the middle cerebral artery, which is more acute in childhood than in the adulthood [9,10]. Other vessels that may be involved are the thalamo-perforating and choroidal arteries. In addition, the lateral perforators make a more acute angle than the medial perforators, which more readily undergo stretching during head trauma. The lateral perforators are shorter in children, encouraging them to stretch. Anatomically, therefore, between the intraparenchymal and the extraparenchymal segment of the lenticulostriate arteries, which are relatively fixed (the first connected to the brain parenchyma and the second to the middle cerebral artery), there is a mobile segment which, if stretched by trauma, results in vasospasm and/or thrombosis, causing ischemia of the tributary territory. Moreover, the sphenoid bone in children is not yet fully developed and does not therefore fully cover the temporal lobes, meaning that the brain has greater mobility than the skull base in case of violent deceleration. This would facilitate stretching of the lenticulostriate arteries by traumatic forces, causing vasospasm and thrombosis [11-13]. Another rare condition which may cause stroke in children is a rare syndrome characterized by diffuse cerebral edema and coma as a result of a mutation of the CACNA1A gene, coding for a structural protein for the calcium channel [14] not present in the case we described. Clearly, the occurrence of a minor head trauma in patients with this syndrome can cause an acute ischemic stroke due to a massive edema. Another pathogenetic theory vive that the friction generated between the lenticulostriate arteries and the brain parenchyma that occurs during the separation of the gray and white matter following a brain trauma in children may cause of vasospasm and thrombosis. This would lead to a shutdown of the lenticulostriate flow causing ischemia in the internal capsule with a subsequent facio-brachio-crural hemiparesis [9]. It has also been shown that certain viral infections, especially varicella zoster [15], can cause secondary vascular disease, thus increaseing susceptibility to develop ment of thrombosis or post-traumatic arterial vasospasm. This parameter was negative in our case because the patient had no clinical history of varicella or herpes.

The clinical history of these lesions is particularly favorable because they are usually small, and also because in a period ranging from several weeks to several months, there is usually a complete remission of facial-brachial-crural hemiparesis, typical of this pathology, despite persistence of the ischemic area on MRI. This is due to the well-known neuronal plasticity of the CNS and in particular of the primary motor cortex [16,17]. The most effective therapeutic approach thus appears to be the conservative one although a review of the pertinent literature over the last 10 years does not clearly indicate which type of treatment is better. In our experience, and in the light of the different pathologies responsible for this type of injury, administration of osmotic diuretics (such as mannitol 0,25-0,50 g/kg 4-6 times a day), to reduce perilesional edema and oral anticoagulants (such as calciparine 80-100 U/kg), to prevent acute stroke and secondary pathologies (emboligen heart disease, thrombophilia and acute traumatic arterial dissections)[18] represent an optimal treatment option in childhood.

## Conclusion

Stroke after a minor head trauma in children under 18 months of age is a very rare condition (< 2% of all ischemic stroke in childhood), and is closely related to the anatomic peculiarities of the brain and skull base in childhood. Young patients should be closely monitored and treated conservatively with osmotic diuretics to reduce perilesional edema and oral anticoagulants to prevent acute stroke. Thanks to neuronal plasticity, deficits generally resolve in a period ranging from 1 week to 3 months. At the same time, however, it is very important to exclude, by means of instrumental and laboratory studies, conditions that could favour the onset of ischemia, including emboligen heart disease, thrombophilia and acute traumatic arterial dissections. Generally, prognosis in these cases is good. In our experience, administration of osmotic diuretics and oral anticoagulant drugs represents an optimal form of treatment.

## Competing interests

The authors declare that they have no competing interests.

## Consent

Written informed consent was obtained from the parents of the patient for publication of this case report and accompanying images.

## Authors' contributions

AL performed the neurosurgical management. NM participated to the neurosurgical management. CM participated in the literature review and in the article's translation. MS participated in the design of the study. RD conceived of the study, and participated in its design and coordination. All authors read and approved the final manuscript.

## References

[B1] Shaffer L Rich PM Pohl KR Ganesan V Can mild head injury cause ischemic stroke? Arch Dis Child 2003 88 3 267 9 10.1136/adc.88.3.267 12598402 PMC1719474

[B2] Haas DC Pineda GS Lourie H Juvenile head trauma syndromes and their relationship to migraine Arch Neurol 1975 32 11 727 30 1180741 10.1001/archneur.1975.00490530049003

[B3] Buompadre MC Arroyo HA Stroke Group Basal ganglia and internal capsule stroke in childhood--risk factors, neuroimaging, and outcome in a series of 28 patients: a tertiary hospital experience J Child Neurol 2009 24 6 685 91 10.1177/0883073808330163 19264737

[B4] Nabika S Kiya K Satoh H Mizoue T Oshita J Kondo H Ischemia of the internal capsule due to mild head injury in a child Pediatr Neurosurg 2007 43 4 312 5 10.1159/000103313 17627149

[B5] Rana KS Behera MD Adhikari KM Ischemic Stroke Following Mild Head Injury: Is it the Cause? Indian Pediatr 2006 43 11 994 7 17151405

[B6] Ruff RM Iverson GL Barth JT Bush SS Broshek DK NAN Policy and Planning Committee Mild Traumatic Brain Injury Committee of the Head Injury Interdisciplinary Special Interest Group of the American Congress of Rehabilitation Medicine: Definition of mild traumatic brain injury J Head Trauma Rehabil 1993 8 86 87 10.1097/00001199-199309000-00008

[B7] Kieslich M Fiedler A Heller C Kreuz W Jacobi G Minor head injury as cause and co-factor in the aetiology of stroke in childhood: a report of eight cases J Neurol Neurosurg Psychiatry 2002 73 1 13 6 10.1136/jnnp.73.1.13 12082038 PMC1757298

[B8] Fullerton HJ Johnston SC Smith WS Arterial dissection and stroke in children Neurology 2001 9;57 7 1155 60 Review 10.1212/wnl.57.7.1155 11601431

[B9] Maki I Akimoto H Enomoto T Injuries of the basal ganglia following head trauma in children Childs Brain 1980 7 113 23 7438836 10.1159/000119936

[B10] Umansky F Gomes FB Dujovny M Diaz FG Ausman JI Mirchandani HG Berman SK The perforating branches of the middle cerebral artery. A microanatomical study J Neurosurg 1985 62 261 268 10.3171/jns.1985.62.2.0261 3968566

[B11] Dharker SR Mittal RS Bhargava N Ischemic lesions in basal ganglia in children after minor head injury Neurosurgery 1993 33 863 865 10.1227/00006123-199311000-00012 8264884

[B12] Martin NA Doberstein C Zane C Caron MJ Thomas K Becker DP Posttraumatic cerebral arterial spasm: transcranial Doppler ultrasound, cerebral blood flow, and angiographic findings J Neurosurg 1992 77 575 583 10.3171/jns.1992.77.4.0575 1527618

[B13] Stein SC Graham DI Chen XH Smith DH Association between intravascular microthrombosis and cerebral ischemia in traumatic brain injury Neurosurgery 2004 54 687 691 10.1227/01.NEU.0000108641.98845.88 15028145

[B14] Kors EE Terwindt GM Vermeulen FL Fitzsimons RB Jardine PE Heywood P Love S van den Maagdenberg AM Haan J Frants RR Ferrari MD Delayed cerebral edema and fatal coma after minor head trauma: role of the CACNA1A calcium channel subunit gene and relationship with familial hemiplegic migraine Ann Neurol 2001 49 6 753 60 10.1002/ana.1031 11409427

[B15] Bodensteiner JB Hille MR Riggs JE Clinical features of vasculopathy associated with primary varicella infection Am J Dis Child 1992 146 100 1736633 10.1001/archpedi.1992.02160130102029

[B16] Kochanek PM Pediatric traumatic brain injury: quo vadis? Dev Neurosci 2006 28 4-5 244 55 Review 10.1159/000094151 16943648

[B17] Anderson V Catroppa C Morse S Haritou F Rosenfeld J Functional plasticity or vulnerability after early brain injury? Pediatrics 2005 116 6 1374 82 10.1542/peds.2004-1728 16322161

[B18] Bernard TJ Goldenberg NA Armstrong-Wells J Amlie-Lefond C Fullerton HJ Treatment of childhood arterial ischemic stroke Ann Neurol 2008 63 6 679 96 10.1002/ana.21406 18496844

